# Antibiotic susceptibility of *Clostridium difficile* is similar worldwide over two decades despite widespread use of broad-spectrum antibiotics: an analysis done at the University Hospital of Zurich

**DOI:** 10.1186/s12879-014-0607-z

**Published:** 2014-11-26

**Authors:** Andrea C Büchler, Silvana K Rampini, Simon Stelling, Bruno Ledergerber, Silke Peter, Alexander Schweiger, Christian Ruef, Reinhard Zbinden, Roberto F Speck

**Affiliations:** Division of Infectious Diseases and Hospital Epidemiology, University Hospital of Zurich, University of Zurich, Raemistrasse 100, Zurich, 8091 Switzerland; Division of Internal Medicine, University Hospital of Zurich, University of Zurich, Raemistrasse 100, Zurich, 8091 Switzerland; Institute of Medical Microbiology, University of Zurich, Gloriastrasse 30/32, Zurich, 8006 Switzerland; Ergon Informatik AG, Kleinstrasse 15, Zürich, 8008 Switzerland; Institute of Medical Microbiology and Hygiene, University of Tübingen, Elfriede-Aulhorn Str. 6, Tübingen, Germany; Internal Medicine, Hospital Schwyz, Waldeggstrasse 10, Schwyz, 6430 Switzerland; Hirslanden Klinik, Witellikerstrasse 40, Zürich, 8032 Switzerland

**Keywords:** Clostridium difficile, Antibiotic susceptibility, Diarrhea, EUCAST

## Abstract

**Background:**

*Clostridium difficile* infection (CDI) remains a major health problem worldwide. Antibiotic use, in general, and clindamycin and ciprofloxacin, in particular, have been implicated in the pathogenesis of CDI. Here, we hypothesized that antibiotics that are highly active *in vitro* against *C. difficile* are less frequently associated with CDI than others. The primary goals of our study were to determine if antibiotic susceptibility and CDI are associated and whether the antimicrobial susceptibility of *C. difficile* changed over the years.

**Methods and results:**

We examined a large panel of *C. difficile* strains collected in 2006-2008 at the University Hospital of Zurich. We found that the antimicrobial susceptibilities to amoxicillin/clavulanate, piperacillin/tazobactam, meropenem, clindamycin, ciprofloxacin, ceftriaxone, metronidazole and vancomycin were similar to those reported in the literature and that they are similar to those reported in other populations over the last two decades. Antibiotic activity did not prevent CDI. For example, thre use of meropenem, which is highly active against all strains tested, was a clear risk factor for CDI. Most of the antibiotics tested also showed a higher minimum inhibitory concentration distribution than that of EUCAST. All strains were susceptible to metronidazole. One strain was resistant to vancomycin.

**Conclusions:**

Antibiotic susceptibilities of the collection of *C. difficile* from the University Hospital of Zurich are similar to those reported by others since the 1980. Patients treated with carbapenems and cephalosporins had the highest risk of developing CDI irrespective of the antimicrobial activity of carbapenems.

**Electronic supplementary material:**

The online version of this article (doi:10.1186/s12879-014-0607-z) contains supplementary material, which is available to authorized users.

## Background

Antibiotic-associated pseudomembranous colitis due to toxin-producing clostridia was first recognized by Bartlett et al. [1]. Today this manifestation is best known as *Clostridium difficile* infection (CDI). CDI is the major cause of nosocomial diarrhea. However, incidence rates for community- vs. hospital-acquired CDI nowadays are similar with 11.16 and 12.1 per 100,000 person years, respectively [2].

*C. difficile* is a Gram-positive spore-forming anaerobic rod that replicates exclusively in the lumen of the gastrointestinal (GI) tract. Disease manifestations are due to enterotoxin A, which affects barrier integrity, and cytotoxin B, which induces cell death [3],[4]. The clinical manifestations of CDI include mild-to-profuse diarrhea with abdominal pain and, in the most severe form, toxic megacolon and perforation. The latter presentation has a mortality rate of 25-40% [5]. The overall mortality rate of CDI increased significantly since 2000 to approximately 6% today [6]. That increase is usually attributed to the emergence of the ribotype 027 strain [6],[7], but its cause is most likely multifactorial [6]. Mutations in the ribotype 027 strain are thought to increase production of toxins A and B [8]. A binary toxin is also produced [9]-[11]. Fatality rates of CDI caused by strains with genes for the binary toxin are twice those of CDI caused by strains that lack the toxin. However, it is unknown if the binary toxin directly contributes to the increased fatality rate or if it is an epiphenomenon of strains with higher virulence [9].

The main risk factor for developing CDI is prior or ongoing antibiotic use [12] that disrupts the flora of the GI tract. *C. difficile* exists in a vegetative form or as a spore. Disruption of the flora of the GI tract enables *C. difficile* to sporulate and the vegetative forms to proliferate, and if they contain the genetic information for toxin formation, to induce CDI.

Various antibiotics have antimicrobial activity against *C. difficile*. However, it is largely unknown whether the inherent activity of those antibiotics protects against CDI. In other words, some antibiotics with activity against *C. difficile* (e.g., metronidazole) may prevent or delay CDI despite disrupting the microbial flora [13],[14]. Loss of activity of fluoroquinolones against *C. difficile* and the associated increase of CDI suggest that antibiotic activity against *C. difficile* may, at least partially, hinder proliferation and toxin production despite disrupting the GI flora [15].

We hypothesized that antibiotics, which are highly active *in vitro* against *C. difficile*, are less frequently associated with CDI than others. Our primary goal was to determine if antibiotic susceptibility and CDI are associated, and thus, we determined the *in vitro* activity of antibiotics against *C. difficile* recovered in 2006-2008 at a tertiary university hospital. Furthermore, the minimum inhibitory concentrations (MICs) were compared with published MICs and the MIC distributions of EUCAST.

## Methods

From September 2006 to June 2007 and from February 2008 to August 2008, all *C. difficile* strains cultured from stool of patients with diarrhea were prospectively collected in the laboratory of the Institute of Medical Microbiology (IMM) at the University of Zurich. The first specimens were collected to evaluate for the presence of the ribotype 027 in Switzerland. The second set was used to compare various assays. Patients were hospitalized or seen on an outpatient basis at the University Hospital of Zurich (USZ), a tertiary care hospital. There were no repeat stool specimens from patients with recurrent CDI. The study was approved by the cantonal ethic committee of Zurich, Department of Health (#EK-1561). The ethics committee waived the need for informed consent from the patients for this retrospective analysis.

Charts were reviewed retrospectively for 94 patients who had a stool specimen positive for culture or toxin. Clinical details were entered into a case report form generated in the database system Access (Microsoft Schweiz GmbH, Wallisellen, Switzerland). The following data were collected: demographics (*e.g*., sex, age and ethnicity), duration of hospitalization, ward, health state (*e.g.*, previous history, drug history with focus on anti-infective, gastric, cytostatic and immunosuppressive medication, procedures, such as surgery, tube feeding and their complications), and microbiological results as related to CDI. “Underlying disease” was defined as the leading diagnosis, and “complication” was defined as a disease engrafted on the lead diagnosis (Additional file 1: Table S1). Because antibiotic treatment may precede development of CDI for up to 8 weeks [13], we recorded all antibiotics for this time frame before collection of the stool specimen.

### Isolation of *C. difficile*

Stool specimens were inoculated onto cycloserin-cefoxitin-fructose agar (CCFA, Difco, Detroit, MI) plates that were incubated under anaerobic conditions for at least 2 days at 37°C. Suspicious colonies were yellowish spreading colonies on CCFA (*i.e.,* acid production from fructose) and were confirmed by conventional methods (*i.e*., gas chromatography of metabolic fatty acids) and an agglutination assay for the *C. difficile* somatic antigen (Microscreen *C. difficile* latex confirmation assay, Lab M Limited, Heywood, UK).

### Detection of *C. difficile*toxin

*C. difficile* toxin testing was conducted in a cytotoxicity assay [16]. Briefly, monolayers of human fibroblasts (MRC-5, courtesy of W. Bossart, Institute of Medical Virology) were maintained in tubes with 2 ml of 10% fetal calf serum in Dulbecco’s minimal essential medium (DMEM, Gibco Life Technologies, Zug, Switzerland) and inoculated with 200 μl of the supernatant of a centrifuged stool suspension (diluted 1:10 in phosphate buffered saline, centrifuged for 10 minutes at 3000 g). After an overnight incubation at 37°C, the monolayers were observed for cytotoxic effects. In cases of positive cytotoxicity but a negative culture for *C. difficile,* the specificity of the cytotoxic effect by *C. difficile* cytotoxin was proved by a neutralization assay. Briefly, supernatants (100 μl) of the centrifuged stool suspensions were preincubated (30’, room temperature) with an equal volume of *C. sordellii* antitoxin (TechLab, Blacksburg, VA) that neutralizes *C. difficile* cytotoxin and, in parallel, with phosphate buffered saline, and both mixtures were retested in the cell-culture cytotoxicity assay. Neutralization of the cytotoxic effect by the antitoxin was considered confirmation of a positive toxin result [16]. From September 2006 to June 2007, all isolates of *C. difficile* were frozen in skim milk and stored at −80°C. From February 2008 to August 2008, isolates were kept in a chopped meat suspension and stored at room temperature. *C. difficile* strains from patients with a negative cytotoxin assay were further characterized by PCR. To detect the toxin genes *tcdA* and *tcdB,* a crude DNA extract was obtained. Toxin genes were detected in a multiplex PCR approach with the primers TA1/TA2 and TB1/TB2 as described [17].

### Antimicrobial susceptibility testing

Antibiotic susceptibility testing was done on subcultures of the isolates of *C. difficile* on brucella sheep blood agar (Difco) with the Etest (Biomérieux, Marcy-l’Etoile, France). Plates were incubated for 48 h under anaerobic conditions [18]. The Etest, based on predefined gradients of antibiotic concentrations on a plastic strip, allowed us to determine the exact MIC of anti-infective drugs. The following antibiotics were selected for susceptibility testing because of their association with CDI: amoxicillin/clavulanate, ceftriaxone, ciprofloxacin, clindamycin, meropenem, metronidazole, piperacillin/tazobactam and vancomycin. The MIC results were categorized in susceptible, intermediate and resistant, following the guidelines of CLSI (Performance Standards for Antimicrobial Susceptibility Testing; 23rd Informational Supplement; http://www.clsi.org/) or, if those were not available, with the values of EUCAST (European Committee on Antimicrobial Susceptibility Testing; http://www.eucast.org/).

### Compilation of literature as related to antibiotic susceptibility of *C. difficile*strains

We used “*Clostridium difficile*” and “susceptibility” as key words to identify articles reporting data on antibiotic susceptibility (MIC50, MIC90 or MIC distributions) of *C. difficile.* We focused our literature research on papers published between 1980 and 2013 since the focus of our work was to get a most comprehensive idea about resistance development over time and to compare our data from Zurich to published data.

### Statistical analysis

Statistical analyses used Stata/SE 13.1 (Stata, College Station, TX). Odds ratios were calculated by comparing the usage of antibiotics in relation to the duration from admission to *C. difficile*–positive testing in the study population to the overall usage of antibiotics at the USZ in 2006-2008. Differences in the MICs between the data from IMM and EUCAST were calculated using the Wilcoxon rank-sum test and the non-parametric equality-of-medians test. The Wilcoxon-rank-sum test determines if the MICs from the two groups are from populations with the same distribution, and the equality-of-medians test tests the null hypothesis that the samples of the two groups were drawn from populations with the same median.

## Results

### Characteristics of the study population

The patient cohort (n = 94) was predominantly of Caucasian origin (i.e., 84% Swiss, 12.8% from the European Community, 1.1% from the U.S. and only 2.1% of Asian origin). Of the patients, 82/94 (87.2%) were inpatients, and 34/92 (37%) underwent surgery. The most common underlying diseases were malignancies and cardiovascular diseases (Additional file 1: Table S1).

Diarrhea was explicitly documented in the charts in 81.9% of the cases. Hospital directives do not indicate testing for *C. difficile* with no evidence of diarrhea. Thus, most likely all patients included in this study suffered from diarrhea even when documentation was missing. Average duration of diarrhea was 6.8 days (median: 3 days). In 46/94 (inpatients: 40, outpatients: 6) stool specimens, *C. difficile* grew in the culture, and its toxin was detected by the cell-culture cytotoxin assay directly from the stool samples (Table 1). In 38/94 (inpatients: 33, outpatients: 5) stool specimens, *C. difficile* grew, but the specimen was negative in the cytotoxin assay. In 17 of those 38 cultures, the toxin was detected at the DNA level by PCR. Also 10/94 specimens (inpatients: 9, outpatients: 1) were culture negative, but had a positive cytotoxin result confirmed by the neutralization test. In five of those specimens with negative culture, another specimen sample taken a couple of days apart from the one analyzed for the *C. difficile* toxin was available, which was then used for antibiotic susceptibility testing.

**Table 1:** **Specimen collection available**

Total number of patients	n = 94							
	"Inpatients" n = 82					"Outpatients" n = 12		
Clinical data sets	Data complete of n = 78			Charts missing n = 4		Data complete		
Stool analysis positive for toxin/culture; n = 94	+/+	−/+	+/−*	+/+	−/+	+/+	−/+	+/−*
	38	31	9	2	2	6	5	1
Antibiotic susceptibility testing done in n = 86	36**	31	4	2	2	6	4**	1

*a stool specimen taken a couple of days apart with growth of *C. difficile* was used for antibiotic susceptibility testing; an alternative stool specimens was only available in 4/9 cases in the inpatient cohort.

**in two and one cases, respectively, from in- and outpatients, cultures were lost and no antimicrobial susceptibility testing could be done.

### Risk factors and clinical presentation

Many risk factors contribute to CDI [19]. Risk factors were evaluated retrospectively before the first positive culture of *C. difficile* (Additional file 1: Table S1). Inpatients were hospitalized for 17.2 days in average (median 9 days). During their current hospitalization, 25 patients (26.6%) were in the ICU for at least 1 day. Thirty patients (32.6%) had one or more complications: 20 patients (21.7%) suffered from infectious complications, and further complications spanned the entire spectrum of medicine (Additional file 1: Table S1). Seven patients (7.4%) died shortly thereafter of causes unrelated to CDI.

In the 8 weeks preceding the first positive culture, 83.3% of the patients (i.e., in- and outpatients) were prescribed antimicrobials, 37.8% antifungal medication, 73.3% gastrointestinal medication, 37.8% immunosuppressive medication, and 20.0% cytostatic medication (Additional file 1: Table S1).

Penicillins (i.e., amoxicillin, amoxicillin/clavulanate and piperacillin/tazobactam) were the most commonly prescribed antibiotics (i.e., 38.5% of our cohort took penicillins with an average duration of 4.6 days (median 4 days), followed by cephalosporins (29.5%) and quinolones (26.9%) with an average duration of 6.8 and 4.9 days, respectively (median 6 and 2 days, respectively)) (Table 2). Further antimicrobials were carbapenems (15.4%), glycopeptides (10.3%), nitromidazoles (5.1%), lincosamides (2.6%), aminoglycosides (3.8%) and others (11.5%). Within the hospitalized patients, 25 were given one class of antibiotics while 34 patients were given more than one class before diagnosis of CDI (Additional file 2: Table S2).

**Table 2:** **Antimicrobial use during hospitalization and prior to detection of** ***Clostridium difficile*** **in stool**

Antimicrobials	Number of patients (%) N = 78†	Duration of treatment	Duration of treatment
		Mean (+/− SD)	Median (range)
**All Antimicrobials**	59 (75.6%)	10.8 (11.0)	7 (1-33)
Penicillins*	30 (38.5%)	4.6 (3.8)	4 (1-12)
Cephalosporins**	23 (29.5%)	6.8 (31.5)	6 (1-26)
Fluoroquinolones***	21 (26.9%)	4.9 (6.6)	2 (1-12)
**Carbapenems#**	12 (15.4%)	9.8 (6.5)	4 (1-21)
**Glycopeptides##**	8 (10.3%)	5 (8.2)	2 (1-25)
**Nitroimidazoles###**	4 (5.1%)	5.8 (4.6)	5 (1-12)
**Aminoglycosides††**	3 (3.8%)	2.7 (1.5)	3 (1-4)
**Clindamycin**	2 (2.7%)	4 (1.4)	4 (3-5)
**Macrolides†††**	1 (1.3%)	3 (0)	3 (3)
**Trimethoprim/Sulfamethoxazole**	9 (11.5%)	4.7 (5.0)	2 (1-14)

†4 charts of inpatients were missing.

The following antibiotics were recorded:

*Penicillins: amoxicillin (n = 2), amoxicillin/clavulanate (n = 13) acid, piperacillin/tazobactam (n = 18).

**Cephalosporins: cefazolin (n = 2), cefepime (n = 12), ceftazidime (n = 1), ceftriaxone (n = 3), cefuroxime (n = 5).

***Fluoroquinolones: ciprofloxacin (n = 19), levofloxacin (n = 1), norfloxacin (n = 1).

#Carbapenems: ertapenem (n = 3), imipenem/cilastatin (n = 2), meropenem (n = 8).

##Glycopeptides: teicoplanin (n = 2), vancomycin (n = 6); all glycopeptides were given intravenously.

###Nitroimidazoles: metronidazole (n = 3), ornidazole (n = 1).

††Aminogylcosides: garamycin (n = 1), tobramycin (n = 2).

†††Macrolides: azithromycin (n = 1).

### Antibiotic susceptibility pattern of *C. difficile*

We observed a uniform susceptibility to metronidazole, meropenem, and piperacillin/tazobactam, with one outlier for vancomycin. The one outlier was a true vancomycin-resistant strain or an erroneous MIC determination. We believe that the observed resistance was a laboratory artifact. Unfortunately, the strain was lost. Over 90% of all strains were susceptible to amoxicillin/clavulanate. In contrast, more than 90% of the strains were intermediate or resistant to clindamycin, ceftriaxone and ciprofloxacin (Table 3).

**Table 3:** **Antimicrobial Susceptibility of CDAD Isolates**

Antimicrobials (n = 86)	MIC interpretative criteria (μg/ml) (CLSI)			Susceptible	Intermediate	Resistant
	S	I	R			
**Amoxicillin/Clavulanate**	≤4/2	8/4	≥16/8	81 (94.2%)	1 (1.2%)	4 (4.7%)
**Ceftriaxone**	≤16	32	≥64	3 (3.5%)	60 (65.1%)	33 (30.4%)
**Ciprofloxacin@**	≤2	4	≥8	0	1 (1.2%)	85 (98.8%)
**Clindamycin**	≤2	4	≥8	7 (8.1%)	34 (39.5%)	45 (52.3%)
**Meropenem**	≤4	8	≥16	86 (100%)	0	0
**Metronidazole¶**	≤8	16	≥32	86 (100%)	0	0
**Piperacillin/Tazobactam**	≤32	64	≥128	86 (100%)	0	0
Vancomycin*	≤2		>2	85 (98.8%)	0	1 (1.2%)

@CLSI and EUCAST did not define breakpoints for ciprofloxacin. Here instead are the breakpoints given for moxifloxacin defined by CLSI which should closely approximate the breakpoints of ciprofloxacin.

¶Breakpoints for metronidazole by EUCAST: S ≤ 2; R2 mg/L.

*No breakpoints defined by CLSI; breakpoints presented by EUCAST.

**Figure 1:**
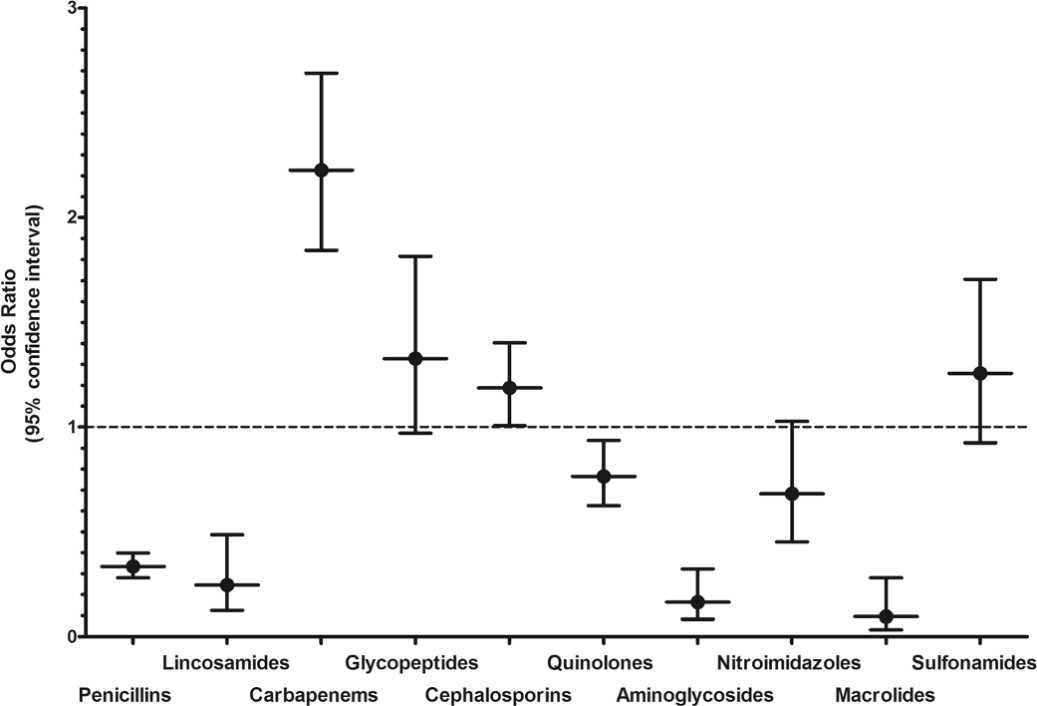
**Patients treated with carbapenems or cephalosporins appear to have had a higher risk for developing CDI.** Odds ratios were calculated by comparing the intake of the various antibiotics examined in our study with the defined daily intake of the antibiotics prescribed in the University Hospital of Zurich during the study period. The dashed line separates the antibiotics which are associated with a significant risk for CDI.

We wondered whether certain anti-infective drugs were over-represented in our patient cohort and, thus, preferentially associated with CDI. We compared the antimicrobial usage of our patient cohort to the overall usage of antimicrobials in the USZ during the same period as defined by daily dose/100 bed days. Carbapenems were given considerably more often to the study population (Figure 1). The numbers of prescriptions for glycopeptides and sulfonamides were about the same in those two groups. On the other side, penicillins, fluoroquinolones, lincosamides, aminoglycosides, and macrolides were prescribed significantly less often than the cumulative usage of the hospital.

The MIC50 and 90 did not reveal a straightforward increase over the last three decades (Additional file 3: Table S3). A closer look, however, showed that the MIC is subtly increasing for amoxicillin/clavulanate, ceftriaxone, piperacillin/tazobactam and metronidazole. The MICs we measured, among all MICs reported, were always at the highest level. In the 1980s, ciprofloxacin and clindamycin already showed a sharp increase in the MIC50 and 90 [20]-[26].

### MIC distribution of *C. difficile*to various antibiotics at the University Hospital of Zurich is similar to data from EUCAST

**Figure 2:**
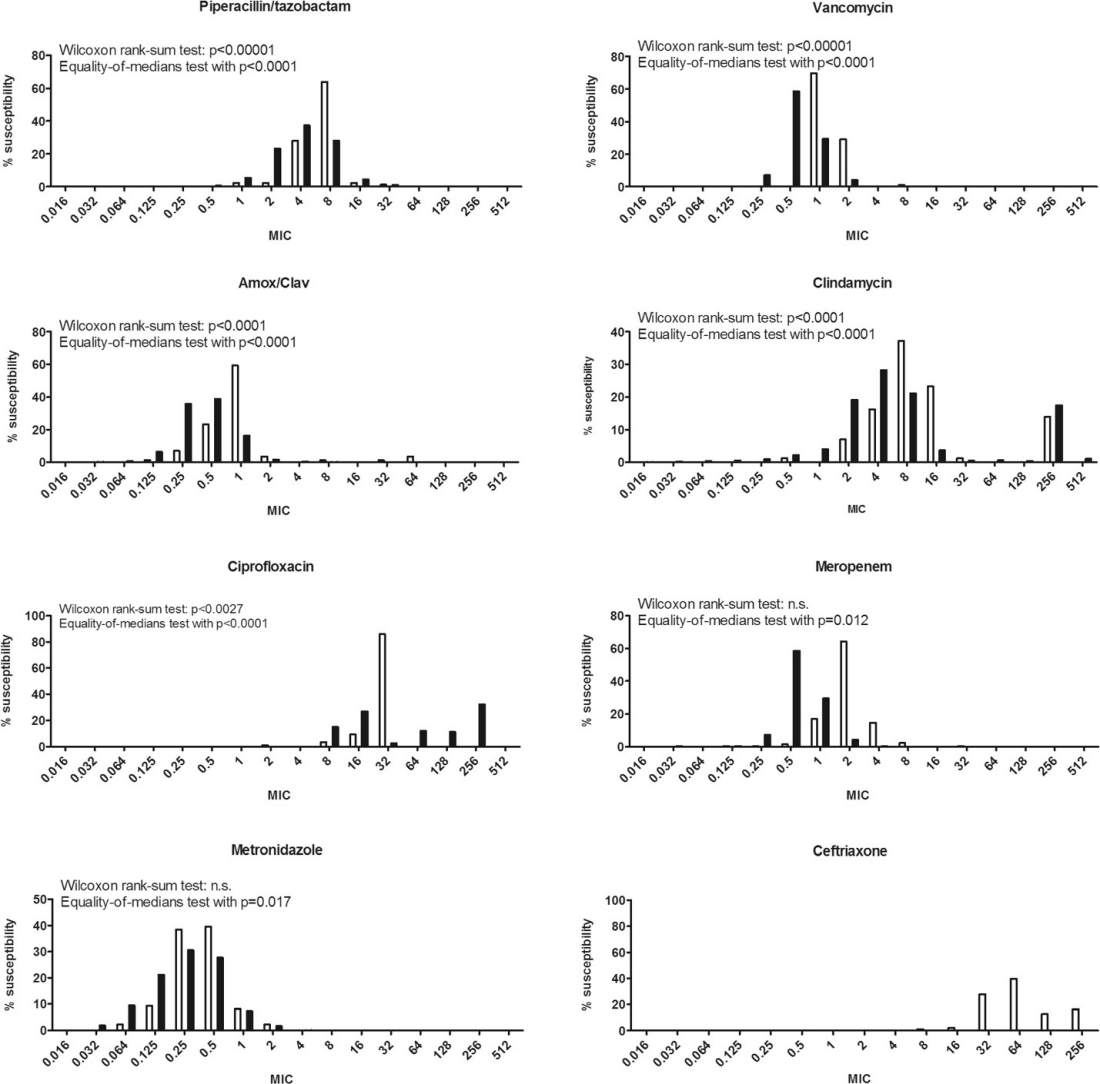
**MIC from the collection of** ***C. difficile*** **from ZH and from EUCAST.** The white bars were the data from ZH, the black bars from EUCAST. We compared the data from ZH with the data from EUCAST with the Wilcoxon rank-sum test and the nonparametric equality-of-medians test.

We compared the MIC distribution of the isolates described here with data from worldwide sources made available by EUCAST (http://mic.eucast.org/Eucast2/). The distributions of the epidemiological cut-off values (ECOFF) showed significant differences between the one obtained by the IMM and EUCAST (Figure 2) by the Wilcoxon rank-sum test for amoxicillin/clavulanate (p < 0.0001), clindamycin C p < 0.0001), ciprofloxacin (p < 0.0027), metronidazole (p = 0.0003), piperacillin/tazobactam (p < 0.00001) and vancomycin (p < 0.00001). Similar results were obtained for the difference of the medians of the two groups using the nonparametric equality-of-medians test (amoxicillin/clavulanate, clindamycin C, piperacillin/tazobactam, ciprofloxacin and vancomycin, p < 0.0001; meropenem, p = 0.012; metronidazole, p = .0.017).

## Discussion

Here, we determined the antibiotics that are associated with CDI in a tertiary hospital center in Switzerland and whether the antibiotic susceptibility of *C. difficile* strains influenced the occurrence of CDI. Our main findings are that i) the antibiotics mostly associated with CDI are carbapenems and cephalosporins, ii) the preserved microbial activity by some antibiotics to *C. difficile* does not prevent CDI, and iii) antibiotic susceptibility showed a trend to increases of the MIC50 and MIC90 of half of the tested antibiotics for the strains collected at the USZ. Very importantly, all *C. difficile* strains were susceptible to metronidazole and vancomycin, except one vancomycin-resistant strain that we believe was a laboratory artifact.

Patients with CDI had a wide variety of underlying diseases (Additional file 1: Table S1). Neoplastic, cardiovascular and gastrointestinal diseases were most common and probably reflect the frequency of hospitalization for these conditions. We observed no accumulation in any distinct division or ward, rendering highly unlikely that a local outbreak contributed to the number of CDI cases.

We made a major effort to assess complications that preceded the CDI. Infectious complications were by far the most frequent and self-explanatory complication as a trigger for CDI. All other complications occurred less frequently and were equally distributed with each representing 1-7% of the total (Additional file 1: Table S1).

Fifteen patients suffered from CDI although they had no antibiotic use in their history. These patients had a number of risk factors: 12/15 had proton pump inhibitors, and 7/15 suffered from malignancies treated with cytostatic and/or immunosuppressive drugs. Only one patient lacked identifiable risk factors. We know that proton pump inhibitors, for example, represents an independent risk factor for CDI [19]. Notably, 34/90 (37.8%) of patients received antifungal drugs. Some studies have reported that *Candida* in the GI-tract acts as protective factor against CDI, and that the use of antifungals drugs is an independent risk factor for CDI [27],[28]. We do not know the extent to which antifungal drugs contributed to the CDI in our cohort.

In our cohort, most cases of CDI occurred in patients treated with penicillins, followed by patients treated with cephalosporins, quinolones and carbapenems. However, we must take into account the rank order of prescribing the various antibiotics. Indeed, the defined daily dose/100 bed days was the highest for penicillins, followed by cephalosporins, quinolones and carbapenems (defined daily dose/100 bed days (DDD/100bd) in 2006, 2007, and 2008 for penicillins were 23.22, 25.77, and 26.10, for cephalosporins were 9.36, 10.45 and 9.67, for quinolones 8.78, 9.82, 9.9, for carbapenems 3.66, 4.45 and 4.08, respectively). Considering the rank order, we identified the administration of carbapenems and cephalosporins as a major risk factor for CDI in this cohort of patients. Glycopeptides and sulfonamides are most likely also a risk factor for CDI; however, the OR for these two antibiotic classes was not statistically significant. In a paradoxical finding, glycopeptides were identified as a potential trigger for CDI. The reason is most likely its co-administration with other broad-spectrum antibiotics and the fact that its intravenous application has no therapeutic role against *C. difficile*. Notably, in 9/11 cases where vancomycin was prescribed, the patients received a combination of antibiotics. It remains unclear whether penicillins (i.e., amoxicillin, amoxicillin/clavulanate and piperacillin/tazobactam) due to their inherent activity against *C. difficile,* and the aminoglycosides and macrolides due to their lack of activity against anaerobes are less likely associated with development of CDI. Our data agree with a recently published meta-analysis that reported the strongest association for cephalosporins and clindamycin with CDI and a lesser one for carbapenems, trimethoprim/sulphonamides, fluoroquinolones and penicillin combinations [29]. The differing results we observed for clindamycin, penicillins and for fluoroquinolones are surprising – it might be due to patient selection in the hospital or genetic differences in the *C. difficile* strains but in fact remains enigmatic.

A major goal was to assess the local susceptibility patterns of the strains isolated (Table 3) and to compare them to patterns of strains worldwide and over the last three decades (Additional file 3: Table S3). We observed uniform susceptibility of *C. difficile* vis-à-vis meropenem, tazobactam/piperacillin, and metronidazole. Only 1/86 isolates was resistant to vancomycin with an MIC measured of 6 μg/ml. This finding was striking since vancomycin resistant isolates are a rarity worldwide. We believe that this resistant strain is a laboratory artifact *i.e.,* erroneous MIC determination. In any case, the reduced vancomycin activity would have no clinical relevance: vancomycin at the doses given perorally or rectally to treat CDI far exceeds the concentration of an MIC of 6 μg/ml. Most isolates were susceptible to amoxicillin/clavulanate. Note that EUCAST has defined MIC clinical breakpoints solely for metronidazole and vancomycin, and CLSI has them for most antibiotics but not for vancomycin and ciprofloxacin. The *C. difficile* strains showed mixed susceptibility to the other three antibiotics tested: the predominant majority of isolates were of intermediate susceptibility or resistant to ceftriaxone and clindamycin; less than 10% were still susceptible. All isolates but one was entirely resistant to ciprofloxacin.

Meropenem’ s antimicrobial activity against *C. difficile* does not appear to protect against CDI; patients on meropenem suffered more frequently from CDI. This is most likely due to the disruption of the GI flora and the overall morbidity of patients requiring carbapenems or co-medications, such as proton pump inhibitors. Very positively, the nitromidazoles and glycopeptides were still close to 100% active against *C. difficile* despite their frequent use.

We compared the local susceptibility pattern to the ECOFF (epidemiological cut-off value) data collection of EUCAST "European Committee on Antimicrobial Susceptibility Testing (http://www.eucast.org). The ECOFF aims to differentiate between wild-type and non-wild-type strains (acquired resistance mutations), based on MIC. Surprisingly, we found that the variance and the median of the MIC from ZH differ from the MICs from EUCAST. Overall, it appeared that the MIC for the antibiotics studied at ZH showed a shift to higher MIC apart from ciprofloxacin and metronidazole. MIC for ciprofloxacin was markedly higher for the data provided by EUCAST than for the data in ZH even though all strains in ZH were resistant. This difference in MIC might be related to ribotype 027, which is known for its particular high resistance to ciprofloxacin. We also observed two populations of strains when looking at the MIC for clindamycin and for amoxicillin/clavulanate. EUCAST does not report this separation in two populations for amoxicillin/clavulanate. The differences noted are most likely due to local prescription habits/directives; indeed, at the University Hospital of Zurich, the directives promote the use of amoxicillin/clavulanate and less of ciprofloxacin whenever appropriate, in particular because of the progressive increase of ciprofloxacin resistance of gram-negative bacteria.

The data collected by EUCAST, however, do not integrate the MIC evolution over time. A compilation of the literature since the first identification of *C. difficile* did not reveal a substantial change of antimicrobial susceptibility over time. In 1980, Dzink and Bartlett reported that over 95% of all strains tested were susceptible to vancomycin, penicillin G, ampicillin, and metronidazole, 60% of the strains were susceptible to clindamycin, and all strains were resistant to the cephalosporins tested (*i.e.,* cephalotin and cefoxitin) [24]. Thus, the discord between the activities of penicillins and cephalosporins was observed already in the very first report. Looking at all data, there is tendency of increases in MIC for all antibiotics tested over the last three decades, but a clear picture is lacking (Additional file 3: Table S3).

Limitations of the study are the number of cases and the retrospective nature of this work. The number of cases we analyzed is in the range of cases presented in published work; nonetheless, we acknowledge that interpretations must be done cautiously due to the limited number of cases and the changes in microbiological assays over time. Our study was further limited by the lack of individual treatment information for the control patients. We therefore had to rely on hospital-wide prescribed DDDs of the different antimicrobials as a comparison group without information on which drugs were given simultaneously. This and the limited sample size precluded multivariable analyses including all antimicrobials which would have been preferable.

## Conclusions

In summary, the antibiotic susceptibilities of the *C. difficile* samples from the University Hospital of Zurich are similar to those reported by others. Patients treated with carbapenems and cephalosporins had the highest risk of developing CDI, irrespective of the antimicrobial activity of carbapenems. Most likely, the underlying diseases or complications, in concert with the carbapenems’ activity on the GI-tract flora, rendered these patients more susceptible to CDI. Differences of MICs between the data from ZH vs EUCAST are minor and are most likely due to local prescriptions habits. Very importantly, all strains, but one, were fully susceptible to metronidazole and vancomycin the antibiotic cornerstone for the treatment of CDI.

## Authors’ contributions

SKR, CR, RZ, SP and RFS designed the study; ACB did all the chart reviews and put the tables and figures together. SKR supervised the chart reviews. AS configured the initial data base for data collection. SS and BL did the statistics. Microbiology work was done by ACB, SP and RZ. ACB wrote the manuscript, SKR, RZ and RFS edited the manuscript. All authors read and approved the final manuscript.

## Additional files

## Electronic supplementary material

Additional file 1: Table S1: Patient characteristics prior to first *C. difficile* positive culture or toxin. (DOCX 36 KB)

Additional file 2: Table S2: Number of inpatients with multiple classes of antibiotics prior to CDI. (DOCX 18 KB)

Additional file 3: Table S3: Susceptibility of C. difficile to various antibiotics over time. (DOCX 125 KB)

Below are the links to the authors’ original submitted files for images.

Authors’ original file for figure 1

Authors’ original file for figure 2
