# Supplementary material for: Antibiotic susceptibility of Clostridium difficile is similar worldwide over two decades despite widespread use of broad-spectrum antibiotics: an analysis done at the University Hospital of Zurich
Source: BMC Infect Dis. 2014 Nov 26;14:607. doi: 10.1186/s12879-014-0607-z (PMC4247760; doi:10.1186/s12879-014-0607-z)
Supplement: Supplementary file 1 — Additional file 1: Table S1: Patient characteristics prior to first C. difficile positive culture or toxin. (DOCX 36 KB) [file 12879_2014_607_MOESM1_ESM.docx]

Supplementary Table 1: Patient characteristics prior to first C. difficile positive culture or toxin

|  | Number of patients  (%) | Mean (+/- SD) | Median (range) |
| --- | --- | --- | --- |
| Total number of patients | 94 |  |  |
| Age (avg±sd): 56.4±17.3 |  |  |  |
| Men | 48 (51.1 %) |  |  |
| Underlying disease |  |  |  |
| Tumor | 36 (38.3 %) |  |  |
| Cardiovascular | 16 (17.0 %) |  |  |
| Urogenital | 12(12.8 %) |  |  |
| Gastrointestinal | 9 (9.6 %) |  |  |
| Infectious | 5 (5.3 %) |  |  |
| Systemic * | 5 (5.3%) |  |  |
| Metabolic | 3 (3.2 %) |  |  |
| Musculoskeletal | 3 (3.2 ) |  |  |
| Lung disease | 2 (2.1 ) |  |  |
| Traumatic | 3 (3.2%) |  |  |
| **In- vs outpatients** |  |  |  |
| Inpatients | 82 (87.2 %) |  |  |
| Outpatients | 12 (12.8 %) |  |  |
| **Days of hospitalization** | 80** (85.1%) | 17.2 (25.7) | 9 (2-58) |
| ICU | 25 (26.6 %)*** | 11.2 (11.6) | 7 (2-29) |
| Surgical ward | 19 (20.2 %) | 9.7 (8.6) | 5 (1-29) |
| Internal medicine ward | 7 (7.4 %) | 13.6 (17.8) | 5 (2-50) |
| **Tube feeding (n=90)§** | 12 (13.2 %) | 20.1 (26) | 11.5 (3-98) |
| Surgery performed (n=92)# | 34 (37.0 %) | 17.2 (24.5) | 9.8 (1-48) |
| Complications (n=92#) | 30 (32.6 %) |  |  |
| Infectious | 20 (21.7 %) |  |  |
| Cardiovascular | 6 (6.5 %) |  |  |
| Lung disease | 6 (6.5 %) |  |  |
| Systemic ¶ | 5 (5.4 %) |  |  |
| Musculosceletal | 4 (4.3 %) |  |  |
| Bleeding | 3 (3.3 %) |  |  |
| Dermatologic | 3 (3.3 %) |  |  |
| Urogenital | 3 (3.3 %) |  |  |
| Gastrointestinal | 2 (2.2 %) |  |  |
| Traumatic | 1 (1.1 %) |  |  |
| Tumor@ | 1 (1.1 %) |  |  |
| Metabolic | 0 |  |  |
| Cases of death (n=94) | 7 (7.4 %) |  |  |
| Medication (n=90 §) |  |  |  |
| Antimicrobials | 75 (83.3 %) |  |  |
| Antifungal | 34(37.8%)§§ |  |  |
| Gastrointestinal | 66 (73.3 %) |  |  |
| Immunosuppressive | 34 (37.8 %) |  |  |
| Cytostatic | 18 (20.0 %) |  |  |
| without any medication | 2 (2.2 %) |  |  |
| without any antimicrobials | 15 (16.7 %) |  |  |

* systemic diseases were hemophilia A, cystic fibrosis (n=2), macrophage activation syndrome and acute renal insufficiency

** 2 patients were diagnosed with CDI at the day of hospitalization; they were not included when calculating number of hospitalization days.

*** one patient was hospitalized at the surgical and medical ICU as well explaining the total number of n=25

§ complete electronic and hard copy charts available from n=90

# information on surgery and complications available on n=92

¶ “systemic” included allergic reactions, shock due to splenic rupture, pancytopenia

@ pancreatic tumour diagnosed during hospitalization ; reason for hospitalization was different

§§ Amphotericin n=5, fluconazole n=13, flucytosine n=1, itraconazole n=10, posaconazole n=1, voriconazole n=4
