# Supplementary material for: Antibiotic susceptibility of Clostridium difficile is similar worldwide over two decades despite widespread use of broad-spectrum antibiotics: an analysis done at the University Hospital of Zurich
Source: BMC Infect Dis. 2014 Nov 26;14:607. doi: 10.1186/s12879-014-0607-z (PMC4247760; doi:10.1186/s12879-014-0607-z)
Supplement: Supplementary file 2 — Additional file 2: Table S2: Number of inpatients with multiple classes of antibiotics prior to CDI. (DOCX 18 KB) [file 12879_2014_607_MOESM2_ESM.docx]

Supplementary Table 2: Number of inpatients with multiple classes of antibiotics prior to CDI

| Class of antibiotics | **Number of patients** |
| --- | --- |
| One class of antibiotics | 25 |
| Two classes of antibiotics | 17 |
| penicillins/trimethoprim-sulfamethoxazole | 4 |
| penicillins/fluoroquinolones | 3 |
| penicillins/cephalosporins | 3 |
| cephalosporins/nitroimidazoles | 2 |
| fluoroquinolones/clindamycin | 2 |
| cephalosporins/glycopeptides | 1 |
| cephalosporins/fluoroquinolones | 1 |
| cephalosporins/trimethoprim-sulfamethoxazole | 1 |
| Three classes of antibiotics | 8 |
| carbapenems/cephalosporins/fluoroquinolones | 2 |
| carbapenems/cephalosporins/penicillins | 1 |
| carbapenems/aminoglycosides/macrolides | 1 |
| cephalosporins/penicillins/glycopeptides | 1 |
| cephalosporins/penicillins/fluoroquinolones | 1 |
| cephalosporins/penicillins/nitroimidazoles | 1 |
| fluoroquinolones/aminoglycosides/glycopeptides | 1 |
| Four classes of antibiotics | 6 |
| cephalosporins/fluoroquinolones/nitroimidazoles/penicillins | 2 |
| cephalosporins/fluoroquinolones/glycopeptides/penicillins | 1 |
| cephalosporins/fluoroquinolones/glycopeptides/trimethoprim-sulfamethoxazole | 1 |
| cephalosporins/carbapenems/glycopeptides/penicillins | 1 |
| carbapenems/fluoroquinolones/aminoglycosides/macrolides | 1 |
| Five classes of antibiotics | 2 |
| carbapenems/fluoroquinolones/cephalosporins/glycopeptides/clindamycin | 1 |
| carbapenems/fluoroquinolones/cephalosporins/penicillins/trimethoprim-sulfamethoxazole | 1 |
| Six classes of antibiotics | 1 |
| carbapenems/fluoroquinolones/cephalosporins/penicillins/glycopeptides/ nitroimidazoles | 1 |
