# Supplementary material for: Antibiotic susceptibility of Clostridium difficile is similar worldwide over two decades despite widespread use of broad-spectrum antibiotics: an analysis done at the University Hospital of Zurich
Source: BMC Infect Dis. 2014 Nov 26;14:607. doi: 10.1186/s12879-014-0607-z (PMC4247760; doi:10.1186/s12879-014-0607-z)
Supplement: Supplementary file 3 — Additional file 3: Table S3: Susceptibility of C. difficile to various antibiotics over time. (DOCX 125 KB) [file 12879_2014_607_MOESM3_ESM.docx]

**Suppl. Table 3:** Susceptibility of C. difficile to various antibiotics over time.

**Amoxicillin/clavulanate acid**

| **Reference** | **Year(s) of collection of specimens** | **n** | **Range** | **MIC 50** | **MIC 90** |
| --- | --- | --- | --- | --- | --- |
| Büchler AC et al. | 2006-2008 | 86 | 0.125-6 | 0.75 | 1 |
| [[1](#_ENREF_1)] | 2003-2006 | 157 | 0.12-1 | 0.5 | 1 |
| [[2](#_ENREF_2)] | 2002-2007 | 43 | 0.094-1.5 | 0.38 | 0.75 |
| [[3](#_ENREF_3)] | 2002 | 73 | 0.047-0.5 | 0.125 | 0.38 |

**Ceftriaxone**

| **Reference** | **Year(s) of collection of specimens** | **n** | **Range** | **MIC 50** | **MIC 90** |
| --- | --- | --- | --- | --- | --- |
| Büchler AC et al. | 2006-2008 | 86 | 8-256 | 48 | 256 |
| [[4](#_ENREF_4)] | 1979-2004 | 179 | n.m.* | 32 | 64 |
| [[5](#_ENREF_5)] | 2005 | 116 | 32-256 | 64 | 64 |
| [[6](#_ENREF_6)] | 1993-1997 | 83 | n.m | 12 | >256 |
| [[7](#_ENREF_7)] | 1999-2000 | 186 | 16-256 | 64 | 64 |
| [[8](#_ENREF_8)] | n.m. | 94 | 4-128 | 32 | 64 |
| [[9](#_ENREF_9)] | 1985 | 42 | 2-64 | 32 | 32 |

*n.m.: not mentioned

**Ciprofloxacin**

| **Reference** | **Year(s) of collection of specimens** | **n** | **Range** | **MIC 50** | **MIC 90** |
| --- | --- | --- | --- | --- | --- |
| Büchler AC et al. | 2006-2008 | 86 | 1.5-32 | 32 | 32 |
| [[1](#_ENREF_1)] | 2003-2006 | 157 | 4->32 | 32 | >32 |
| [[10](#_ENREF_10)] | 2005-2010 | 403 | 0.5-128 | 16 | 64 |
| [[11](#_ENREF_11)] | 2004-2006 | 330 | 0.5-32 | 32 | 32 |
| [[12](#_ENREF_12)] | 1993-2007 | 606 | >32 | >32 | >32 |
| [[6](#_ENREF_6)] | 1993-1997 | 83 | n.m. | >32 | >32 |
| [[13](#_ENREF_13)] | n.m. | 50 | 8-128 | 8 | 32 |
| [[14](#_ENREF_14)] | 1995-1996 | 44 | 3->32 | >32 | >32 |
| [[15](#_ENREF_15)] | n.m. | 98 | 2-128 | 8 | 8 |
| [[16](#_ENREF_16)] | n.m. | 100 | 8-32 | 16 | 32 |
| [[9](#_ENREF_9)] | 1985 | 37 | 8 | 8 | 8 |

**Clindamycin**

| **Reference** | **Year(s) of collection of specimens** | **n** | **Range** | **MIC 50** | **MIC 90** |
| --- | --- | --- | --- | --- | --- |
| Büchler AC et al. | 2006-2008 | 86 | 0.5-256 | 8 | 256 |
| [[1](#_ENREF_1)] | 2003-2006 | 157 | 0.25->32 | >32 | >32 |
| [[10](#_ENREF_10)] | 2005-2010 | 403 | 0.06->256 | 8 | >256 |
| [[11](#_ENREF_11)] | 2004-2006 | 330 | 0.047-256 | 8 | 256 |
| [[17](#_ENREF_17)] | 2001-2009 | 113 | 0.06-≥256 | 4 | ≥256 |
| [[2](#_ENREF_2)] | 2002-2007 | 43 | 0.016->256 | 6 | >256 |
| [[12](#_ENREF_12)] | 1993-2007 | 606 | 0.5->256 | 4 | >256 |
| [[4](#_ENREF_4)] | 1979-2004 | 179 | n.m. | 16 | 16 |
| [[18](#_ENREF_18)] | 2005 | 83 | ≥32 | ≥32 | ≥32 |
| [[5](#_ENREF_5)] | 2005 | 116 | 0.5-≥32 | 8 | 16 |
| [[19](#_ENREF_19)] | 2002-2003 | 79 | 0.023-≥256 | 4 | 256 |
| [[20](#_ENREF_20)] | 2000-2001 | 238 | 1->256 | 4 | >256 |
| [[6](#_ENREF_6)] | 1993-1997 | 83 | n.m. | 4 | >256 |
| [[21](#_ENREF_21)] | n.m. | 36 | 0.19->256 | >256 | >256 |
| [[22](#_ENREF_22)] | n.m. | 65 | 0.25-3 | 1 | 2 |
| [[22](#_ENREF_22)] | n.m. | 28 | 0.25->256 | >256 | >256 |
| [[7](#_ENREF_7)] | 1999-2000 | 186 | ≤2->128 | 8 | 16 |
| [[23](#_ENREF_23)] | 1986-2001 | 192 | 2-≥256 | 6 | ≥256 |
| [[3](#_ENREF_3)] | 2002 | 73 | 0.16->256 | 4 | >256 |
| [[14](#_ENREF_14)] | 1995-1996 | 44 | 2->256 | >256 | >256 |
| [[24](#_ENREF_24)] | 1988-1989 | 85 | >256 | >256 | >256 |
| [[24](#_ENREF_24)] | 1988-1989 | 46 | <4->256 | ≤ 4 | >256 |
| [[15](#_ENREF_15)] | n.m. | 98 | <1->128 | 4 | >128 |
| [[8](#_ENREF_8)] | n.m. | 145 | 0.25-≥1024 | 8 | ≥1024 |
| [[25](#_ENREF_25)] | 1985-1986 | 100 | 0.5-128 | 2 | 8 |
| [[9](#_ENREF_9)] | 1985 | 42 | 4-32 | 8 | 32 |
| [[26](#_ENREF_26)] | 1984 | 48 | 0.25->128 | >128 | >128 |
| [[27](#_ENREF_27)] | 1981 | 79 | 0.78->100 | 3.13 | >100 |
| [[28](#_ENREF_28)] | 1980 | 84 | <1->128 | ≤1 | 128 |

**Meropenem**

| **Reference** | **Year(s) of collection of specimens** | **n** | **Range** | **MIC 50** | **MIC 90** |
| --- | --- | --- | --- | --- | --- |
| Büchler AC et al. | 2006-2008 | 86 | 0.38-4 | 1.5 | 2 |
| [[29](#_ENREF_29)] | 2001-2009 | 113 | 0.03-8 | 2 | 4 |
| [[2](#_ENREF_2)] | 2002-2007 | 43 | 0.003-32 | 0.5 | 4 |
| [[3](#_ENREF_3)] | 2002 | 73 | 0.032-1.0 | 0.75 | 1 |
| [[30](#_ENREF_30)] | 1983-2004 | 110 | 1-4 | 2 | 2 |

**Metronidazole**

| **Reference** | **Year(s) of collection of specimens** | **n** | **Range** | **MIC 50** | **MIC 90** |
| --- | --- | --- | --- | --- | --- |
| Büchler AC et al. | 2006-2008 | 86 | 0.064-2 | 0.25 | 0.75 |
| [[1](#_ENREF_1)] | 2003-2006 | 157 | 0.06-1 | 0.25 | 0.5 |
| [[10](#_ENREF_10)] | 2005-2010 | 403 | ≤0.03-4 | 0.5 | 0.5 |
| [[17](#_ENREF_17)] | 2001-2009 | 113 | 0.03-4 | 0.5 | 1 |
| [[11](#_ENREF_11)] | 2004-2006 | 330 | 0.016-1.5 | 0.064 | 0.19 |
| [[31](#_ENREF_31)] | 2002-2007 | 43 | 0.016-2 | 0.19 | 1 |
| [[12](#_ENREF_12)] | 1993-2007 | 606 | <0.016-2 | 0.25 | 0.5 |
| [[4](#_ENREF_4)] | 1979-2004 | 179 | n.m. | <0.5 | <0.5 |
| [[5](#_ENREF_5)] | 2005 | 116 | ≤0.5-4 | 1 | 2 |
| [[30](#_ENREF_30)] | 1983-2004 | 110 | 0.03-0.5 | 0.125 | 0.25 |
| [[20](#_ENREF_20)] | 2000-2001 | 238 | 0.032-8 | 0.125 | 0.25 |
| [[6](#_ENREF_6)] | 1993-1997 | 83 | n.m. | 0.19 | 0.38 |
| [[7](#_ENREF_7)] | 1999-2000 | 186 | 0.5-4 | 1 | 2 |
| [[23](#_ENREF_23)] | 1986-2001 | 192 | 0.06-2 | 0.125 | 0.25 |
| [[3](#_ENREF_3)] | 2002 | 73 | 0.023-0.19 | 0.094 | 0.19 |
| [[14](#_ENREF_14)] | 1995-1996 | 44 | 0.032-0.5 | 0.19 | 0.38 |
| [[8](#_ENREF_8)] | n.m. | 137 | ≤0.06-8 | 0.25 | 2 |
| [[15](#_ENREF_15)] | n.m. | 98 | <1.0 | <1 | <1 |
| [[25](#_ENREF_25)] | 1985-1986 | 100 | 0.064-0.125 | 0.125 | 0.125 |
| [[9](#_ENREF_9)] | 1985 | 37 | 0.25-0.5 | 0.5 | 0.5 |
| [[26](#_ENREF_26)] | 1984 | 42 | <0.12-0.25 | ≤0,12 | 0.25 |
| [[27](#_ENREF_27)] | 1981 | 79 | <0.2-0.39 | 0.2 | 0.39 |
| *[[28](#_ENREF_28)] | 1980 | 84 | <1->128 | ≤1 | ≤1 |

* * 1 Stamm 2, 1 Stamm >128

**Piperacillin-tazobactam**

| **Reference** | **Year(s) of collection of specimens** | **n** | **Range** | **MIC 50** | **MIC 90** |
| --- | --- | --- | --- | --- | --- |
| Büchler AC et al. | 2006-2008 | 86 | 1-24 | 6 | 8 |
| [[1](#_ENREF_1)] | 2003-2006 | 157 | 1-32 | 8 | 16 |
| [[31](#_ENREF_31)] | 2002-2007 | 43 | 0.016-24 | 3 | 6 |
| [[6](#_ENREF_6)] | 1993-1997 | 83 | n.m. | 1 | 3 |
| [[3](#_ENREF_3)] | 2002 | 73 | 0.047-6 | 2 | 3 |
| [[14](#_ENREF_14)] | 1995-1996 | 44 | 3/4-256/4 | 16/4 | >256/4 |

**Vancomycin**

| **Reference** | **Year(s) of collection of specimens** | **n** | **Range** | **MIC 50** | **MIC 90** |
| --- | --- | --- | --- | --- | --- |
| Büchler AC et al. | 2006-2008 | 86 | 0.75-6 | 1 | 1.5 |
| [[1](#_ENREF_1)] | 2003-2006 | 157 | 0.12-2 | 0.5 | 1 |
| [[10](#_ENREF_10)] | 2005-2010 | 403 | 0.06-4 | 0.5 | 1 |
| [[17](#_ENREF_17)] | 2001-2009 | 113 | 0.25-4 | 0.5 | 1 |
| [[11](#_ENREF_11)] | 2004-2006 | 330 | 0.023-2 | 0.5 | 1 |
| [[31](#_ENREF_31)] | 2002-2007 | 43 | 0.25-2 | 0.5 | 1.5 |
| [[12](#_ENREF_12)] | 1993-2007 | 606 | 0.25-8 | 0.5 | 1 |
| [[4](#_ENREF_4)] | 1979-2004 | 179 | n.m. | 1 | 2 |
| [[5](#_ENREF_5)] | 2005 | 116 | 1-4 | 2 | 4 |
| [[30](#_ENREF_30)] | 1983-2004 | 110 | 0.06-4 | 1 | 1 |
| [[20](#_ENREF_20)] | 2000-2001 | 236 | 0.5-8 | 1 | 1 |
| [[6](#_ENREF_6)] | 1993-1997 | 83 | n.m. | 0.25 | 0.5 |
| [[7](#_ENREF_7)] | 1999-2000 | 186 | 0.5-4 | 1 | 2 |
| [[23](#_ENREF_23)] | 1986-2001 | 192 | 0.06-4 | 0.75 | 1 |
| [[3](#_ENREF_3)] | 2002 | 73 | 0.125-3 | 0.5 | 0.75 |
| [[14](#_ENREF_14)] | 1995-1996 | 44 | 0.25-1.5 | 0.5 | 1 |
| [[8](#_ENREF_8)] | n.m. | 138 | ≤0.125-4 | 1 | 4 |
| [[15](#_ENREF_15)] | n.m. | 98 | <0.25-8 | 2 | 4 |
| [[25](#_ENREF_25)] | 1985-1986 | 100 | 0.25-16 | 0.5 | 1 |
| [[26](#_ENREF_26)] | 1984 | 42 | 0.25-1 | 0.5 | 0.5 |
| [[27](#_ENREF_27)] | 1981 | 79 | 0.78-1.56 | 1.56 | 1.56 |
| [[28](#_ENREF_28)] | 1980 | 84 | <1 - 8 | ≤1 | 2 |

**References:**

1. Kunishima H, Chiba J, Saito M, Honda Y, Kaku M: **Antimicrobial susceptibilities of Clostridium difficile isolated in Japan**. *J Infect Chemother* 2013, **19**(2):360-362.

2. Jamal W, Shahin M, Rotimi VO: **Surveillance and trends of antimicrobial resistance among clinical isolates of anaerobes in Kuwait hospitals from 2002 to 2007**. *Anaerobe* 2010, **16**(1):1-5.

3. Jamal WY, Mokaddas EM, Verghese TL, Rotimi VO: **In vitro activity of 15 antimicrobial agents against clinical isolates of Clostridium difficile in Kuwait**. *Int J Antimicrob Agents* 2002, **20**(4):270-274.

4. Taori SK, Hall V, Poxton IR: **Changes in antibiotic susceptibility and ribotypes in Clostridium difficile isolates from southern Scotland, 1979-2004**. *J Med Microbiol* 2010, **59**(Pt 3):338-344.

5. Mutlu E, Wroe AJ, Sanchez-Hurtado K, Brazier JS, Poxton IR: **Molecular characterization and antimicrobial susceptibility patterns of Clostridium difficile strains isolated from hospitals in south-east Scotland**. *J Med Microbiol* 2007, **56**(Pt 7):921-929.

6. Samore MH, Venkataraman L, DeGirolami PC, Merrigan MM, Johnson S, Gerding DN, Carmeli Y, Harbarth S: **Genotypic and phenotypic analysis of Clostridium difficile correlated with previous antibiotic exposure**. *Microb Drug Resist* 2006, **12**(1):23-28.

7. Drummond LJ, McCoubrey J, Smith DG, Starr JM, Poxton IR: **Changes in sensitivity patterns to selected antibiotics in Clostridium difficile in geriatric in-patients over an 18-month period**. *J Med Microbiol* 2003, **52**(Pt 3):259-263.

8. Wust J, Hardegger U: **Studies on the resistance of Clostridium difficile to antimicrobial agents**. *Zentralbl Bakteriol Mikrobiol Hyg A* 1988, **267**(3):383-394.

9. Chow AW, Cheng N, Bartlett KH: **In vitro susceptibility of Clostridium difficile to new beta-lactam and quinolone antibiotics**. *Antimicrob Agents Chemother* 1985, **28**(6):842-844.

10. Liao CH, Ko WC, Lu JJ, Hsueh PR: **Characterizations of clinical isolates of clostridium difficile by toxin genotypes and by susceptibility to 12 antimicrobial agents, including fidaxomicin (OPT-80) and rifaximin: a multicenter study in Taiwan**. *Antimicrob Agents Chemother* 2012, **56**(7):3943-3949.

11. Pituch H, Obuch-Woszczatynski P, Wultanska D, Nurzynska G, Harmanus C, Banaszkiewicz A, Radzikowski A, Luczak M, van Belkum A, Kuijper E: **Characterization and antimicrobial susceptibility of Clostridium difficile strains isolated from adult patients with diarrhoea hospitalized in two university hospitals in Poland, 2004-2006**. *J Med Microbiol* 2011, **60**(Pt 8):1200-1205.

12. Noren T, Alriksson I, Akerlund T, Burman LG, Unemo M: **In vitro susceptibility to 17 antimicrobials of clinical Clostridium difficile isolates collected in 1993-2007 in Sweden**. *Clin Microbiol Infect* 2010, **16**(8):1104-1110.

13. Wilcox MH, Fawley W, Freeman J, Brayson J: **In vitro activity of new generation fluoroquinolones against genotypically distinct and indistinguishable Clostridium difficile isolates**. *J Antimicrob Chemother* 2000, **46**(4):551-556.

14. Cheng SH, Chu FY, Lo SH, Lu JJ: **Antimicrobial susceptibility of Clostridium difficile by E test**. *J Microbiol Immunol Infect* 1999, **32**(2):116-120.

15. Clabots CR, Shanholtzer CJ, Peterson LR, Gerding DN: **In vitro activity of efrotomycin, ciprofloxacin, and six other antimicrobials against Clostridium difficile**. *Diagn Microbiol Infect Dis* 1987, **6**(1):49-52.

16. Delmee M, Avesani V: **Comparative in vitro activity of seven quinolones against 100 clinical isolates of Clostridium difficile**. *Antimicrob Agents Chemother* 1986, **29**(2):374-375.

17. Lin YC, Huang YT, Tsai PJ, Lee TF, Lee NY, Liao CH, Lin SY, Ko WC, Hsueh PR: **Antimicrobial susceptibilities and molecular epidemiology of clinical isolates of Clostridium difficile in taiwan**. *Antimicrob Agents Chemother* 2011, **55**(4):1701-1705.

18. Spigaglia P, Barbanti F, Mastrantonio P, Brazier JS, Barbut F, Delmee M, Kuijper E, Poxton IR: **Fluoroquinolone resistance in Clostridium difficile isolates from a prospective study of C. difficile infections in Europe**. *J Med Microbiol* 2008, **57**(Pt 6):784-789.

19. Pituch H, Brazier JS, Obuch-Woszczatynski P, Wultanska D, Meisel-Mikolajczyk F, Luczak M: **Prevalence and association of PCR ribotypes of Clostridium difficile isolated from symptomatic patients from Warsaw with macrolide-lincosamide-streptogramin B (MLSB) type resistance**. *J Med Microbiol* 2006, **55**(Pt 2):207-213.

20. Aspevall O, Lundberg A, Burman LG, Akerlund T, Svenungsson B: **Antimicrobial susceptibility pattern of Clostridium difficile and its relation to PCR ribotypes in a Swedish university hospital**. *Antimicrob Agents Chemother* 2006, **50**(5):1890-1892.

21. Martirosian G, Szczesny A, Cohen SH, Silva J, Jr.: **Isolation of non-toxigenic strains of Clostridium difficile from cases of diarrhea among patients hospitalized in hematology/oncology ward**. *Pol J Microbiol* 2004, **53**(3):197-200.

22. Bendle JS, James PA, Bennett PM, Avison MB, Macgowan AP, Al-Shafi KM: **Resistance determinants in strains of Clostridium difficile from two geographically distinct populations**. *Int J Antimicrob Agents* 2004, **24**(6):619-621.

23. Ackermann G, Degner A, Cohen SH, Silva J, Jr., Rodloff AC: **Prevalence and association of macrolide-lincosamide-streptogramin B (MLS(B)) resistance with resistance to moxifloxacin in Clostridium difficile**. *J Antimicrob Chemother* 2003, **51**(3):599-603.

24. Johnson S, Samore MH, Farrow KA, Killgore GE, Tenover FC, Lyras D, Rood JI, DeGirolami P, Baltch AL, Rafferty ME *et al*: **Epidemics of diarrhea caused by a clindamycin-resistant strain of Clostridium difficile in four hospitals**. *N Engl J Med* 1999, **341**(22):1645-1651.

25. Nord CE, Lindmark A, Person I: **Susceptibility of anaerobic bacteria to FCE 22101**. *Antimicrob Agents Chemother* 1987, **31**(5):831-833.

26. Gianfrilli P, Luzzi I, Pantosti A, Occhionero M: **In vitro susceptibility of Clostridium difficile isolates to 12 antimicrobial agents**. *Chemioterapia* 1984, **3**(1):41-44.

27. Nakamura S, Nakashio S, Mikawa M, Yamakawa K, Okumura S, Nishida S: **Antimicrobial susceptibility of Clostridium difficile from different sources**. *Microbiol Immunol* 1982, **26**(1):25-30.

28. Dzink J, Bartlett JG: **In vitro susceptibility of Clostridium difficile isolates from patients with antibiotic-associated diarrhea or colitis**. *Antimicrob Agents Chemother* 1980, **17**(4):695-698.

29. Kalos M, Levine BL, Porter DL, Katz S, Grupp SA, Bagg A, June CH: **T cells with chimeric antigen receptors have potent antitumor effects and can establish memory in patients with advanced leukemia**. *Sci Transl Med* 2011, **3**(95):95ra73.

30. Hecht DW, Galang MA, Sambol SP, Osmolski JR, Johnson S, Gerding DN: **In vitro activities of 15 antimicrobial agents against 110 toxigenic clostridium difficile clinical isolates collected from 1983 to 2004**. *Antimicrob Agents Chemother* 2007, **51**(8):2716-2719.

31. Jamal W, Rotimi VO, Brazier J, Duerden BI: **Analysis of prevalence, risk factors and molecular epidemiology of Clostridium difficile infection in Kuwait over a 3-year period**. *Anaerobe* 2010, **16**(6):560-565.
