# Supplementary figures and images for: Antibiotic susceptibility of Clostridium difficile is similar worldwide over two decades despite widespread use of broad-spectrum antibiotics: an analysis done at the University Hospital of Zurich
Source: BMC Infect Dis. 2014 Nov 26;14:607. doi: 10.1186/s12879-014-0607-z (PMC4247760; doi:10.1186/s12879-014-0607-z)

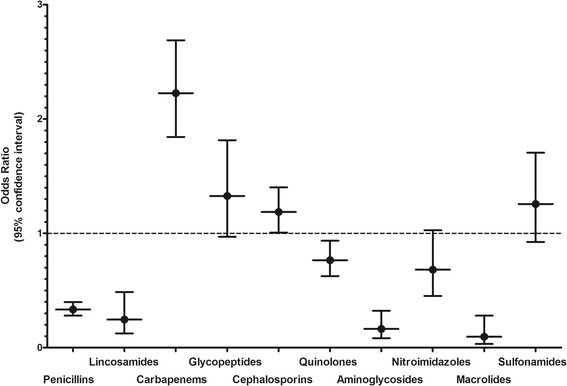

Supplement: Supplementary file 4 — Authors’ original file for figure 1 [file 12879_2014_607_MOESM4_ESM.gif]

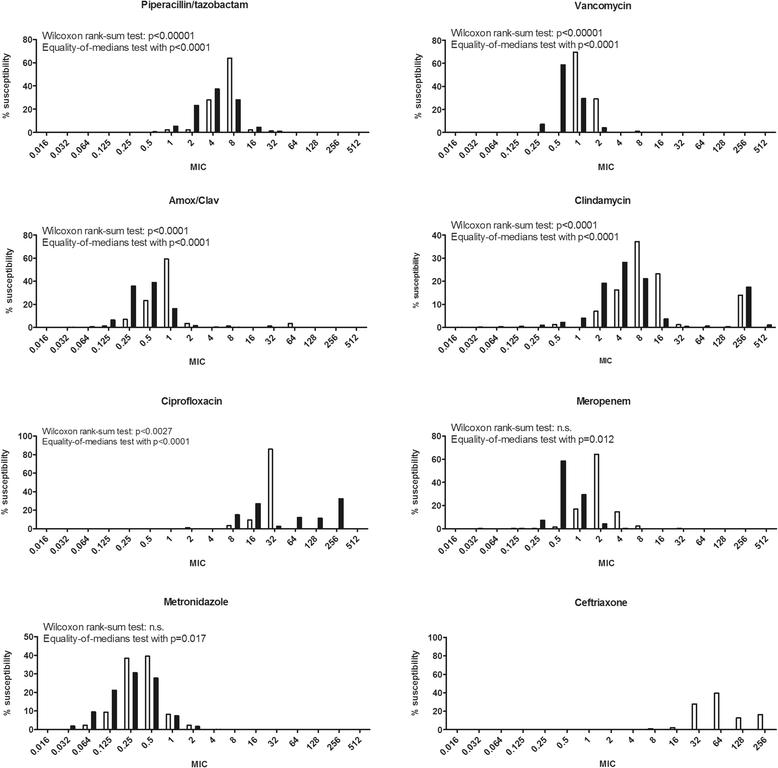

Supplement: Supplementary file 5 — Authors’ original file for figure 2 [file 12879_2014_607_MOESM5_ESM.gif]
